# Integrated multi-omics profiling to establish an IGFBP-based prognostic score for pancreatic ductal adenocarcinoma: unraveling prognostic biomarkers, immune microenvironment crosstalk, and therapeutic implications

**DOI:** 10.3389/fimmu.2025.1600527

**Published:** 2025-05-15

**Authors:** Xiao Guan, Yongrun Mu, Xin Jin, Chengfeng Wang

**Affiliations:** State Key Lab of Molecular Oncology and Department of Pancreatic and Gastric Surgery, National Cancer Center/National Clinical Research Center for Cancer/Cancer Hospital, Chinese Academy of Medical Sciences and Peking Union Medical College, Beijing, China

**Keywords:** pancreatic ductal adenocarcinoma, IGFBP, prognosis, multi-omics analysis, tumor microenvironment

## Abstract

**Background:**

Pancreatic ductal adenocarcinoma (PDAC) is accompanied by endocrine dysfunction, particularly involving dysregulation of the insulin and insulin-like growth factor (IGF) signaling pathways. Clinical manifestations such as hyperglycemia and insulin resistance are common and have been linked to aberrant expression of insulin-like growth factor-binding proteins (IGFBPs). However, the specific roles and mechanisms of IGFBP family genes in PDAC remain unclear.

**Method:**

We conducted a multi-dimensional integrative analysis using publicly available PDAC cohorts, stratifying patients based on IGFBP gene expression profiles. A prognostic model was constructed to classify patients into risk groups. To explore the biological mechanisms underlying IGFBP involvement in PDAC, we further incorporated single-cell transcriptomic sequencing and spatial transcriptomic data to investigate the relationship between IGFBP expression and the tumor immune microenvironment.

**Result:**

Our prognostic model effectively stratified PDAC patients into distinct risk categories with significant survival differences. High-risk patients demonstrated specific IGFBP expression patterns associated with aggressive tumor biology. Single-cell and spatial transcriptomic analyses revealed that IGFBP family genes modulate immune cell infiltration and spatial immune heterogeneity within the tumor microenvironment.

**Conclusion:**

This study identified the IGFBP family genes as key modulators of PDAC progression and immune landscape remodeling. These findings supported the potential of IGFBP family genes as prognostic biomarkers and therapeutic targets, offering new insights into PDAC biology and opportunities for personalized treatment strategies.

## Introduction

Pancreatic ductal adenocarcinoma (PDAC) is one of the most lethal malignancies, characterized by a high mortality rate and rapid progression (1–4). Current projections estimate that PDAC will become the second leading cause of cancer-related deaths worldwide by 2030 (5–7). A major clinical challenge lies in the fact that most PDAC patients are diagnosed at advanced stages, often beyond the optimal window for surgical intervention, rendering curative resection infeasible (8). The disease’s insidious onset, absence of early symptoms, aggressive invasiveness, and high metastatic potential significantly hinder early diagnosis and effective treatment (9). Compounding the difficulty, PDAC shows limited responsiveness to immunotherapy and inherent resistance to radiotherapy, contributing to poor clinical outcomes and dismal prognosis (10).

Currently, carcinoembryonic antigen (CEA) and carbohydrate antigen 19-9 (CA19-9) are the most commonly used clinical biomarkers for pancreatic cancer. However, their limited sensitivity and specificity, especially for early-stage disease, reduce their diagnostic utility (11). These markers often remain within normal ranges during the early phases of PDAC and only exhibit abnormal elevations at advanced disease stages (12). Thus, to improve the prognosis and overall quality of life for PDAC patients, it is imperative to investigate the molecular mechanisms underlying disease progression and to identify novel therapeutic targets and biomarkers.

The pancreas, uniquely composed of both endocrine and exocrine compartments (13, 14), exhibits anatomical and functional integration through overlapping blood supply and paracrine signaling (15, 16). Accumulating evidence suggests that interactions between these two systems may play a pivotal role in PDAC initiation and progression (17–19). For instance, in obese individuals, pancreatic β-cells have been shown to secrete cholecystokinin, which promotes tumor development (16). Conversely, PDAC cells release adrenomedullin-rich exosomes that induce β-cell apoptosis, contributing to endocrine dysfunction (20). Additionally, endocrine-disrupting compounds and hormones secreted by pancreatic endocrine cells have been implicated in the pathogenesis of PDAC (17, 21).

Among these molecular mediators, the insulin-like growth factor-binding protein (IGFBP) family has emerged as a crucial modulator. IGFBPs influence tumor growth, metastasis, and therapeutic resistance through both IGF-dependent and IGF-independent pathways, thereby contributing to PDAC progression (22, 23). These findings underscore the biological complexity of PDAC and highlight the potential importance of the IGFBP family in tumor biology.

In this study, we conducted a comprehensive bioinformatics analysis of IGFBP family genes in PDAC. Based on IGFBP expression profiles, we established novel molecular subtypes of PDAC and stratified patients into risk-based categories. Furthermore, we integrated data from the tumor immune microenvironment, revealing immunological mechanisms potentially influenced by IGFBP activity. Taken together, our findings provide new insights into the role of IGFBPs in PDAC and lay the groundwork for developing more precise diagnostic tools and therapeutic strategies for this highly aggressive cancer.

## Methods

### Data collection

Gene expression profiles and clinical data for PDAC were obtained from The Cancer Genome Atlas (TCGA) and GSE62452 datasets. All non-PDAC pathological subtypes were excluded. Gene expression data were standardized, and patients lacking complete survival or clinical information were removed from the analysis. GSE202051 and GSE235315 were used for single-cell RNA sequencing and spatial transcriptomics analyses and were sourced from the Gene Expression Omnibus database.

### Clustering analysis based on IGFBP family genes

Seven IGFBP family genes were selected for analysis. Patients were stratified into molecular subgroups using a consensus unsupervised clustering algorithm implemented in the “ConsensusClusterPlus” R package. We determined the optimal number of clusters using the elbow method. Clusters with high intra-group correlation and low inter-group correlation were retained for further analyses.

### Multi-omics characterization of IGFBP-based subtypes

To validate the clustering results, Principal Component Analysis (PCA) was performed. Clinical characteristics were compared across subtypes, followed by Kaplan-Meier survival analysis to assess prognostic differences. The tumor microenvironment (TME) was analyzed across subtypes using various immune-related scores, and box plots were generated to visualize TME score distributions.

### Differential analysis and functional enrichment

Differentially expressed genes (DEGs) between subtypes were identified. Functional enrichment analyses, including Gene Ontology (GO), Kyoto Encyclopedia of Genes and Genomes (KEGG), and Gene Set Variation Analysis (GSVA), were conducted to explore the biological differences among subtypes.

### Clustering based on prognostic genes and risk model construction

Univariate Cox regression was applied to screen for prognostically relevant DEGs. Clustering analysis was repeated using these genes, and survival differences were reassessed. Least Absolute Shrinkage and Selection Operator (LASSO) regression was applied in the TCGA cohort to construct a prognostic model, which was validated using the GSE62452 cohort. Patients were divided into high-risk and low-risk groups based on the median risk score, and their association with IGFBP-based subtypes was evaluated.

### Multi-omics analysis of the prognostic model

We assessed correlations between risk scores and clinical variables, and further analyzed TME characteristics using immune cell infiltration data. Patients were classified into immune subtypes to explore the relationship between risk score and immune phenotype. Tumor mutation burden (TMB) was calculated using somatic mutation data, and survival analyses were conducted after stratifying patients by TMB and risk score. The “pRRophetic” package was used to estimate the half-maximal inhibitory concentration (IC50) of various chemotherapeutic agents across the risk groups (24, 25).

### Nomogram construction and validation

A prognostic nomogram integrating clinical variables and risk scores was developed. Calibration curves, receiver operating characteristic (ROC) curves, and decision curve analysis (DCA) were used to assess the nomogram’s predictive performance and clinical utility.

### Single-cell and spatial transcriptomic analyses

For single-cell analysis, we used the Scanpy toolkit (26). Genes expressed in fewer than three cells and cells with <200 or >10,000 genes, fewer than 1000 total counts, or >20% mitochondrial/ribosomal content were excluded. SCVI (https://github.com/scverse/scvi-tools) was used to correct for batch effects. IGFBP gene expression levels were visualized across cell types using bar plots.

For spatial transcriptomic data, the Seurat package was used for preprocessing, including normalization, feature selection, dimensionality reduction, and clustering. Multimodal Intersection Analysis (MIA) was employed to infer cell type distributions across tissue regions. Cell types were annotated based on differentially expressed genes in each cluster (27). CellChat was used to infer cell-cell communication networks (28).

### Statistical analysis

Continuous variables were expressed as means ± standard deviation and compared using Student’s t-test or Mann-Whitney U test. Univariate and multivariate Cox regression analyses were conducted to identify independent prognostic factors, with variables showing P < 0.05 in univariate analysis entered into multivariate models. Hazard ratios (HRs), 95% confidence intervals (CIs), and P-values were reported. Nomogram performance was evaluated using ROC, calibration plots, and DCA. All statistical analyses were conducted using R software (version 4.3.2), and P < 0.05 was considered statistically significant.

#### Multi-color immunohistochemistry

Briefly, 4μm FFEP slides was de-paraffinized in the 100% ethanol (2×2min),95% ethanol (2×2min) and rinsed in distilled water, and immersed in citrate buffer after heat-induced epitope retrieval. After incubation of BSA. the AlphaTSA Multiplex IHC Kit (AXT36100031, AlphaX) was used for staining according to the manufacturer’s Guidelines. The primary antibody and the matching secondary antibody coupled with horseradish peroxidase (HRP) were incubated on the slides. Primary antibodies were IGFBP3 (Cell Signaling, Cat<ns/> <ns/>64143, 1:800), CD8 (ZSGB-BIO, Cat<ns/> <ns/> ZA-0508, 1:600), CD68 (Abcam, Cat<ns/> <ns/> ab192847, 1:200, α-SMA (Cell Signaling, Cat<ns/> <ns/>19245, 1:1200) and PANCK (Abcam, Cat<ns/> <ns/> ab7753, 1:200). Fluorescent images were collected using the ZEISS Axioscan7 microscope and analyzed by ZEISS ZEN (v3.2). The Cancer Hospital Chinese Academy of Medical Science granted approval for the research.

### CD8+ T cell migration assays

PANC-1 with IGFBP3-knockdown generated via siRNA (Santa cruz biotechnology, sc-39587) were cultured in DMEM supplemented with 10% fetal bovine serum under standard conditions (37°C, 5% CO2). When cells reached 70% confluency, the culture medium was replaced with fresh serum-free DMEM. After 12 hours of incubation, the conditioned medium (CM) was collected and filtered through a 0.45μm sterile filter to remove cellular debris. The filtered CM was added to the lower chamber of a 24-well Transwell system (Corning Inc., pore size 8 μm). CD8^+^ T cells were resuspended in RPMI-1640 medium at a density of 2×10^5^ cells/200 μL and seeded into the upper chamber. The plate was incubated for 24 hours under standard conditions to allow chemotactic migration toward CM-derived factors. Migrated cells on the lower membrane surface were fixed with 4% methanol-free formaldehyde (Beyotime Biotechnology) for 30 minutes at room temperature. Fixed cells were stained with 1% ammonium oxalated crystal violet (Solarbio) for 30 minutes, followed by three washes with PBS to remove excess dye.

## Results

**Supplementary Figure S1** showed the flowchart of this study.

### Classification of PDAC into immunologically distinct subtypes based on IGFBP-based clustering analysis

**Figure 1:**
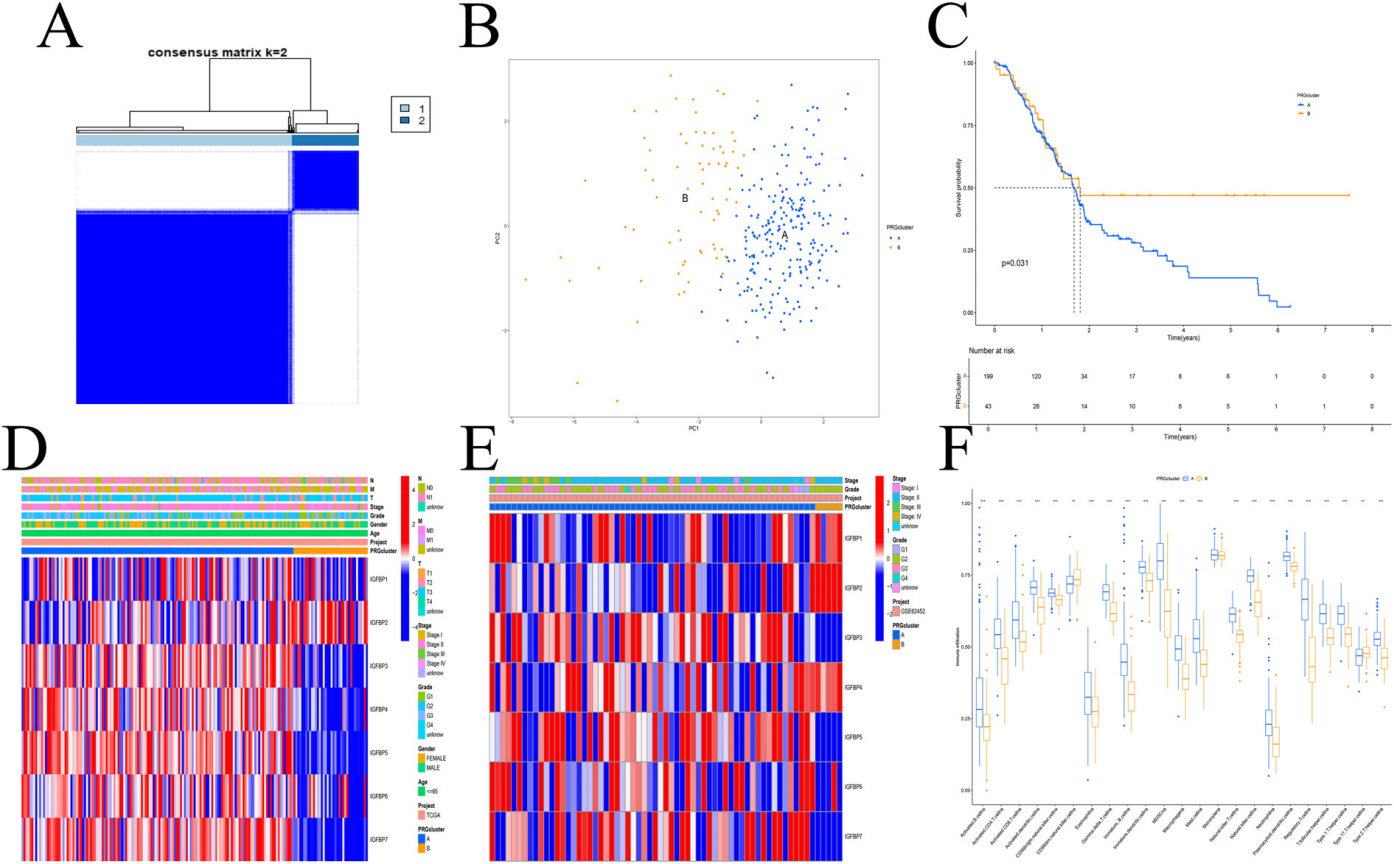
IGFBP subtypes and clinical evaluation. **(A)** Two subtypes and their associated regions. **(B)** PCA analysis. There was a significant difference between the two subtypes. **(C)** Survival analysis. Subtype A had a poorer prognosis. **(D, E)** There was no difference in clinical factors between the two subtypes in the TCGA **(D)** and GEO **(E)** datasets. **(F)** The two subtypes differed significantly in the invasion of immune cells. **p < 0.01; ***p < 0.001.

A total of 309 PDAC patients from the GSE62452 and TCGA cohorts were included for further analysis. Using a consensus clustering approach based on IGFBP family gene expression, we identified two distinct molecular subtypes (**Supplementary Figure S2**), with the optimal number of clusters determined to be k=2 (**Figure 1A**). These subtypes were designated as Subtype A and Subtype B. PCA confirmed a clear separation between the two subtypes (**Figure 1B**). Kaplan–Meier survival analysis revealed that patients in Subtype A had significantly longer overall survival than those in Subtype B (**Figure 1C**). However, no significant differences in clinicopathological variables were observed between the subtypes (**Figures 1D, E**).

**Figure 2:**
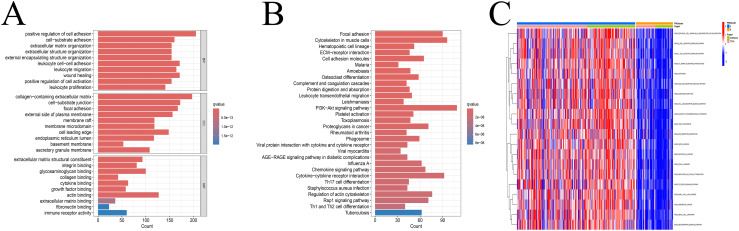
Enrichment analysis. **(A)** GO enrichment analysis revealed that these genes are primarily associated with extracellular matrix, membranous organelle, and binding protein. **(B)** KEGG enrichment analysis indicated that these genes were mainly related to the ECM−receptor interaction, protein processing and cell adhesion. **(C)** GSVA enrichment analysis demonstrated that these genes were predominantly involved in the immune cell signaling pathway and apoptosis. ***p < 0.001.

We next assessed immune cell infiltration characteristics between the subtypes. Subtype A exhibited a higher level of immune cell infiltration compared to Subtype B (**Figure 1F**). GO enrichment analysis showed that DEGs between the subtypes were mainly associated with extracellular matrix organization, membrane-bound organelles, and protein binding functions (**Figure 2A**). KEGG analysis indicated enrichment in pathways related to cell adhesion, protein processing, and ECM-receptor interactions (**Figure 2B**). GSVA revealed enrichment in apoptosis-related and immune signaling pathways (**Figure 2C**).

### Subtype re-classification based on differentially expressed genes

**Figure 3:**
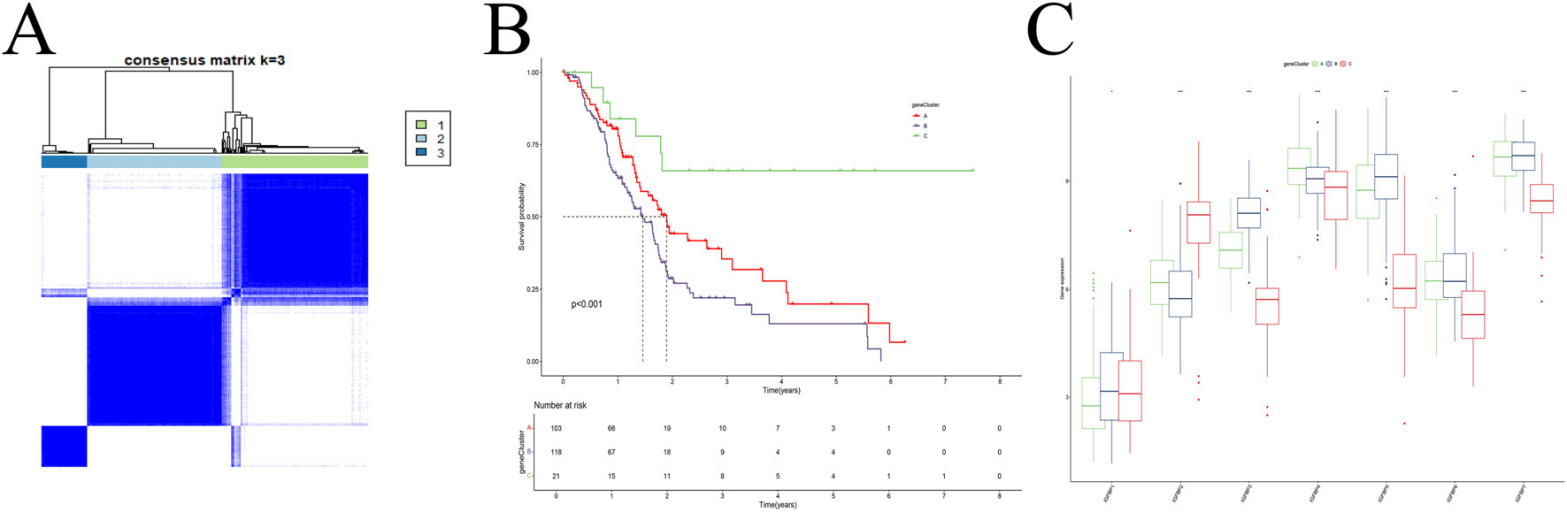
Cluster analysis. **(A)** Dividing patients into three subtypes was optimal. **(B)** Survival analysis. **(C)** The IGFBP family genes exhibit significant differences among the various subtypes.

Univariate and differential expression analyses identified 1303 DEGs among the IGFBP-related subtypes. Using these genes, we re-clustered the patients with the same consensus clustering method (**Supplementary Figure S3**), determining that three clusters (k=3) were optimal (**Figure 3A**). These new gene expression-based subtypes also showed significant survival differences (**Figure 3B**). Notably, the expression patterns of IGFBP family genes varied significantly among the three subtypes (**Figure 3C**).

### Construction and evaluation of the prognostic model

**Figure 4:**
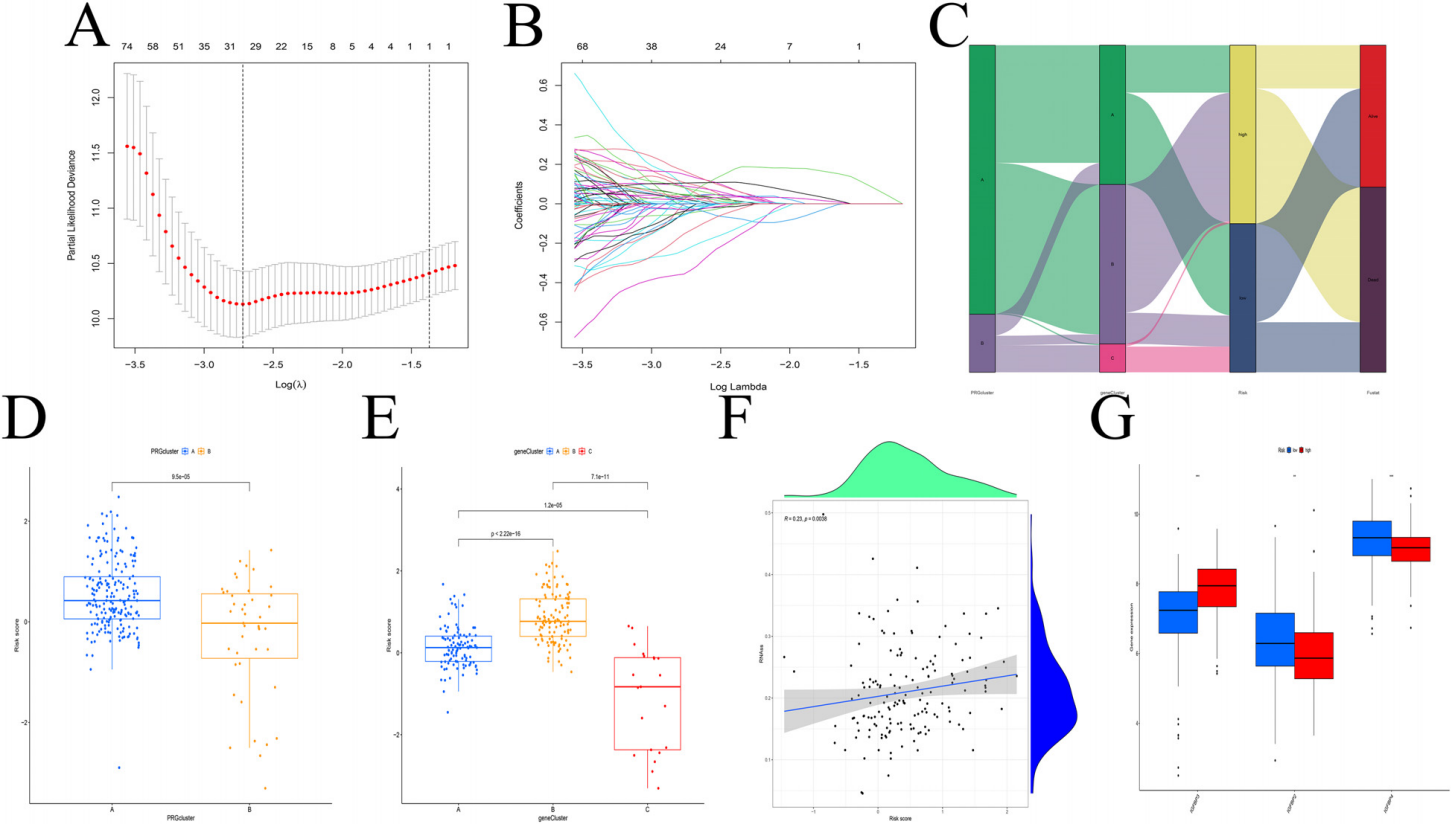
Construction of the predictive model. **(A, B)** LASSO regression analysis. **(C)** Distribution of different subtypes, risk groups, and survival outcomes. **(D, E)** Risk score distribution across different subtypes. **(F)** RNAss values are positively correlated with risk scores. **(G)** Relationship between IGFBP family genes and risk scores. **p < 0.01; ***p < 0.001.

Eight prognostic genes were selected using LASSO regression (**Figures 4A, B**). Based on the median risk score calculated from the TCGA cohort, patients were stratified into high- and low-risk groups. **Figure 4C** shows the distribution of patient risk scores, IGFBP subtypes, gene expression-based subtypes, and survival status. Subtype A (from IGFBP-based clustering) was enriched in high-risk patients, while Subtype B (from gene expression-based clustering) had lower risk scores (**Figures 4D, E**). A positive correlation was observed between risk scores and RNAss values (**Figure 4F**), and IGFBP3 expression was positively associated with higher risk scores (**Figure 4G**).

**Figure 5:**
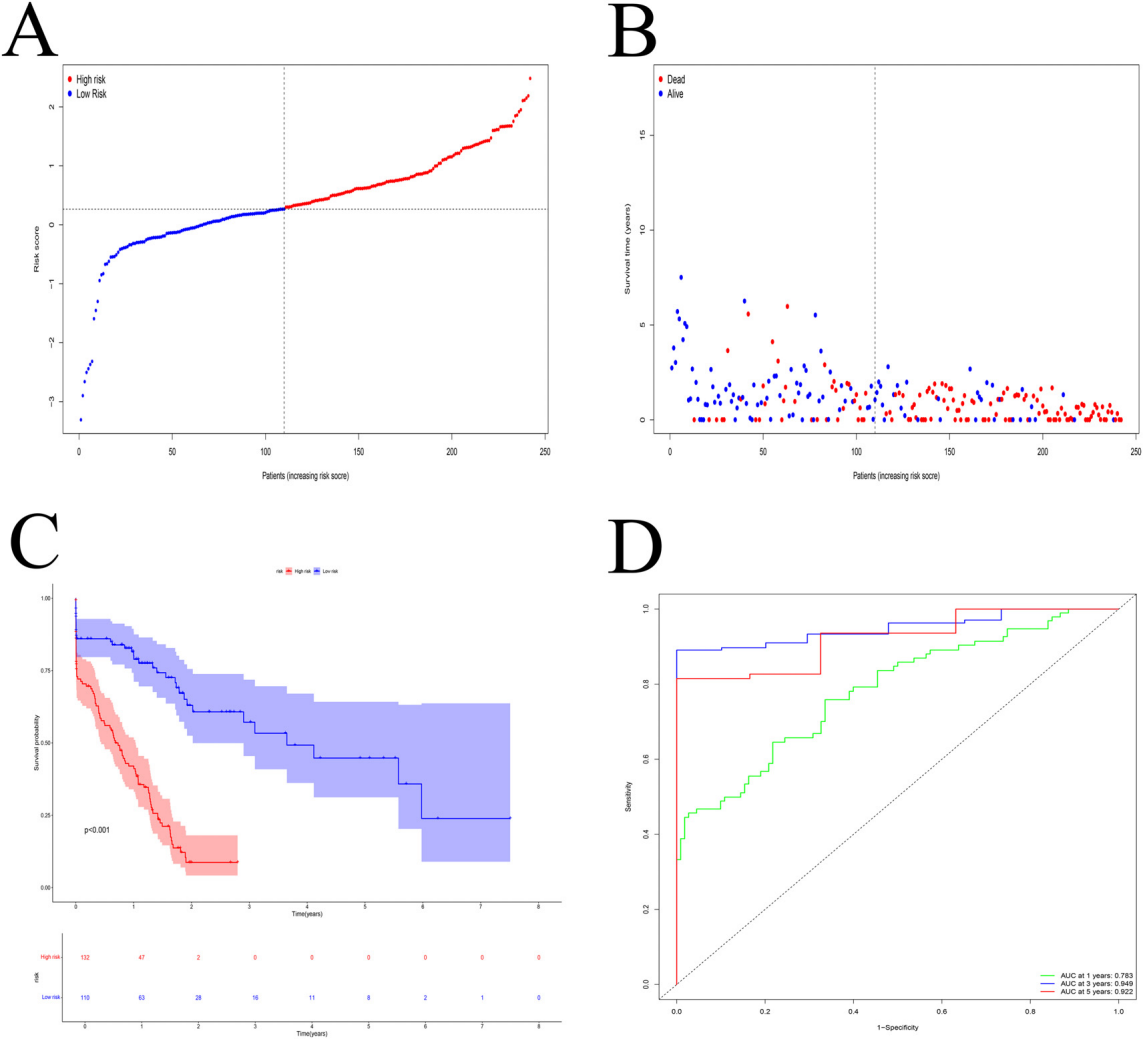
Model evaluation. **(A)** The median risk score was used to categorize the patients into high- and low-risk groups. **(B)** The percentage of PDAC patients who passed away rose in tandem with the risk value. **(C)** Patients with high-risk scores had a poorer prognosis. **(D)** ROC curve. The 1-, 3-, and 5-year AUC were 0.783, 0.949, and 0.922, respectively.

Survival analysis demonstrated that the high-risk group had significantly worse overall survival than the low-risk group (**Figure 5A**). A higher proportion of deaths was observed in the high-risk group (**Figure 5B**). Time-dependent ROC analysis showed strong predictive accuracy of the model, with AUCs of 0.783, 0.949, and 0.922 at 1, 3, and 5 years, respectively (**Figure 5D**).

### IGFBP-based prognostic score shows immune landscape and tumor mutational burden analysis of PDAC

**Figure 6:**
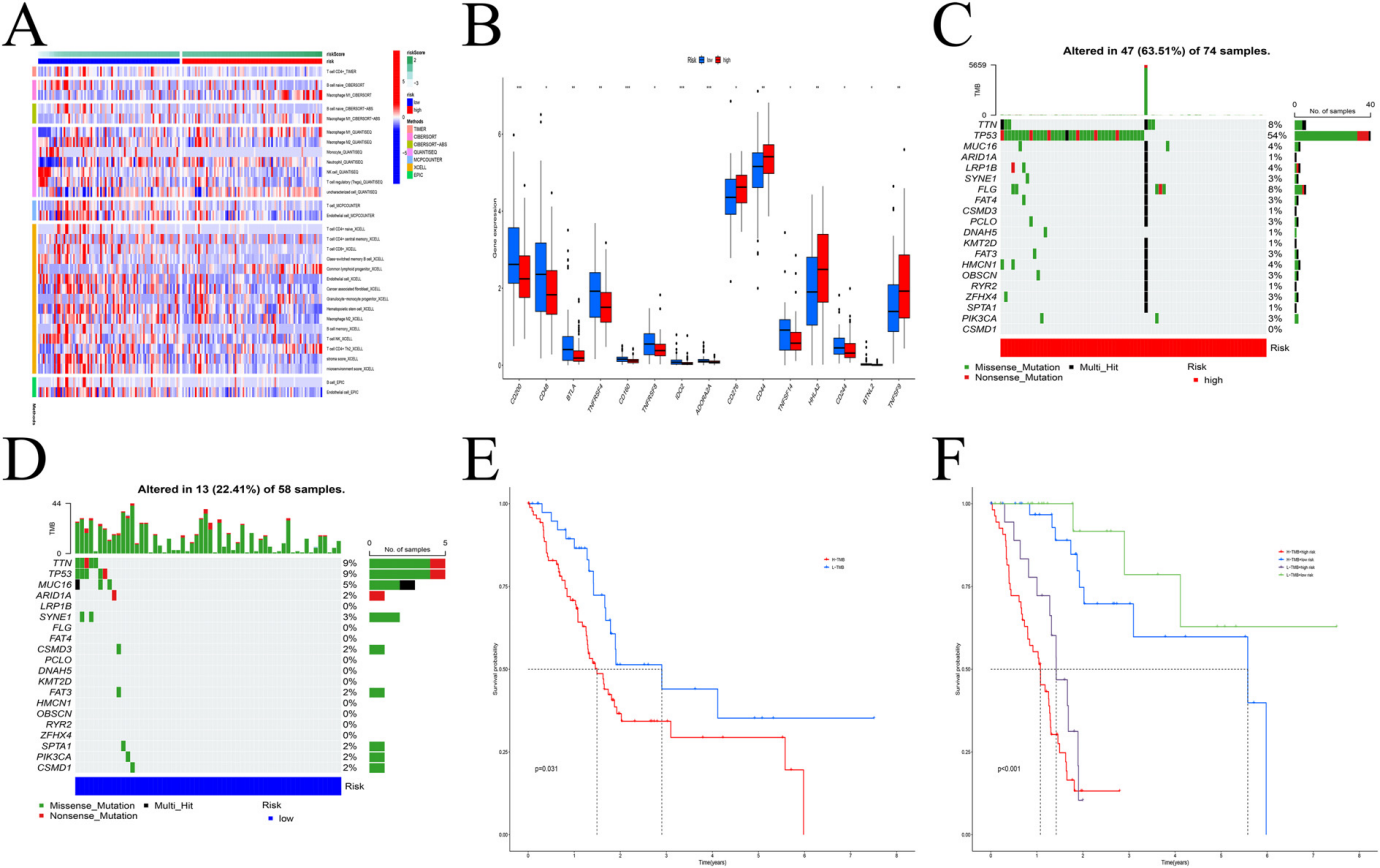
Model multi-omics analysis. **(A)** The infiltration levels of immune cells exhibited significant differences. **(B)** Variations in checkpoint gene expression levels between the two groups. **(C, D)** The frequency of gene mutations in the high **(C)** and low **(D)** risk groups. **(E, F)** Survival analysis. H-TMB had a poor prognosis. * p < 0.05; ** p < 0.01; *** p < 0.001.

Immune correlation analysis revealed that risk scores were significantly associated with the infiltration of various immune cells, including macrophages, T cells, B cells, and dendritic cells (**Figure 6A**, **Supplementary Figure S4**). Differential expression of immune checkpoint genes between the risk groups further highlighted distinct immune microenvironmental features (**Figure 6B**). TMB analysis identified TP53, TTN, and MUC1 as the most frequently mutated genes in both groups (**Figures 6C, D**). Survival analysis indicated that patients with low TMB and low-risk scores had the best prognosis (**Figures 6E, F**).

### Drug sensitivity analysis

**Figure 7:**
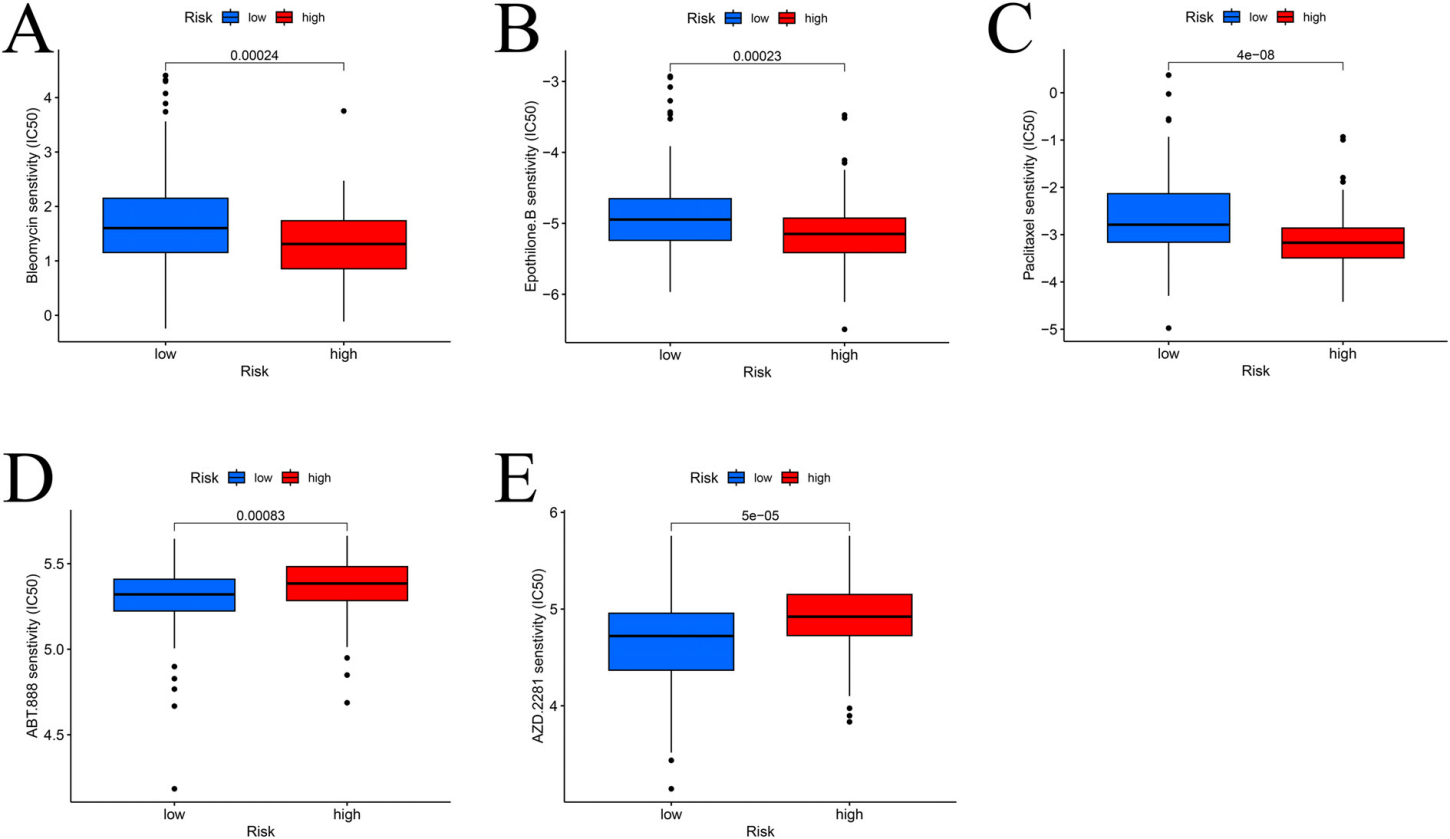
Drug sensitivity analysis. Sensitive drugs in high **(A–C)** and low **(D, E)** risk groups.

To identify potential therapeutic agents, drug sensitivity analysis was performed. High-risk patients were more sensitive to paclitaxel, epothilone B, and bleomycin (**Figures 7A–C**), while low-risk patients showed greater sensitivity to olaparib and veliparib (**Figures 7D, E**).

### Nomogram development and validation

**Figure 8:**
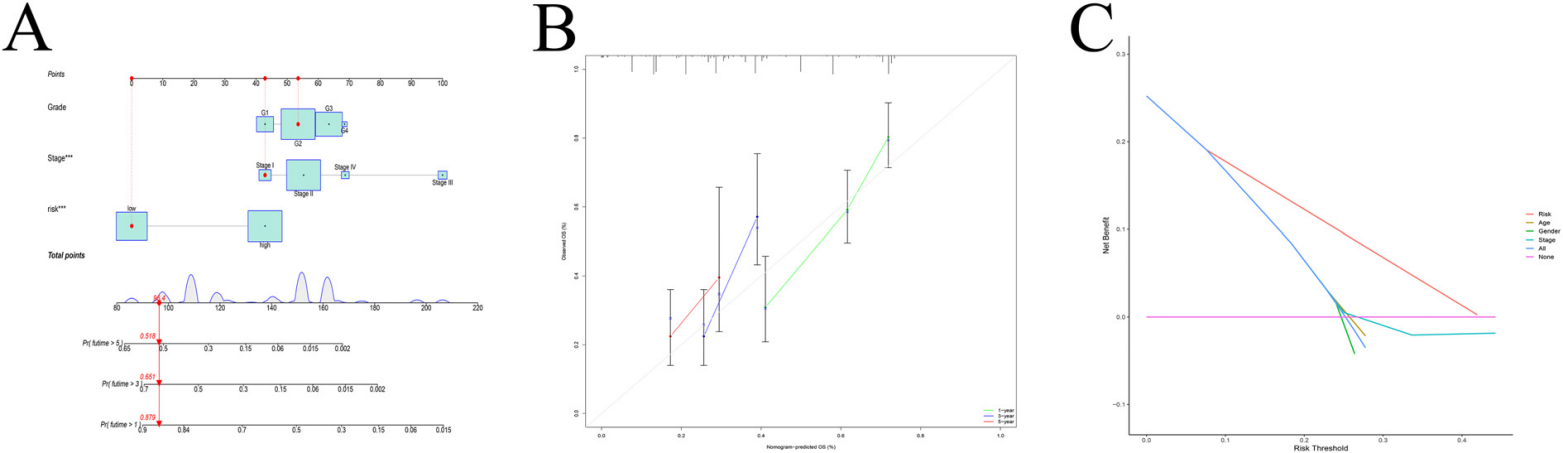
Nomogram construction and evaluation. **(A)** Nomogram. **(B)** Calibration curve. **(C)** DCA curve.

A nomogram integrating clinical variables and risk scores was constructed (**Figure 8A**). Calibration curves demonstrated strong agreement between predicted and observed outcomes (**Figure 8B**), and decision curve analysis confirmed the clinical utility of the nomogram (**Figure 8C**).

### Integration of single-cell and spatial transcriptomic analysis shows the spatiotemporal mapping of IGFBP family in PDAC unveils IGFBP3 hubs driving immune evasion

**Figure 9:**
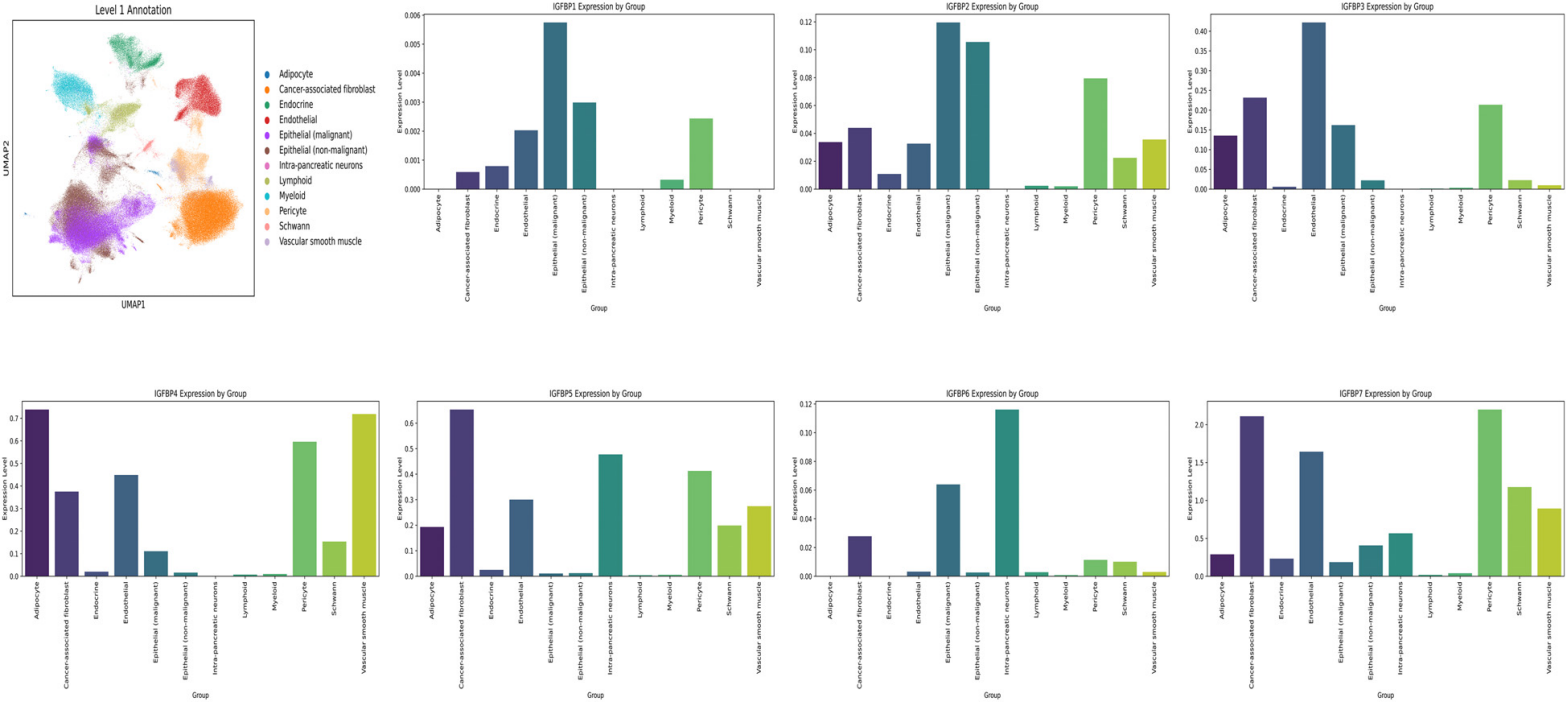
Single cell analysis. Cell Annotation and expression of IGFBP family genes in 12 cell types.

Using the GSE202051 dataset, we analyzed untreated single-cell RNA sequencing data. After batch normalization, malignant epithelial cells were found to express elevated levels of most IGFBP genes, except IGFBP5 and IGFBP7 (**Figure 9**, **Supplementary Figure S5**).

**Figure 10:**
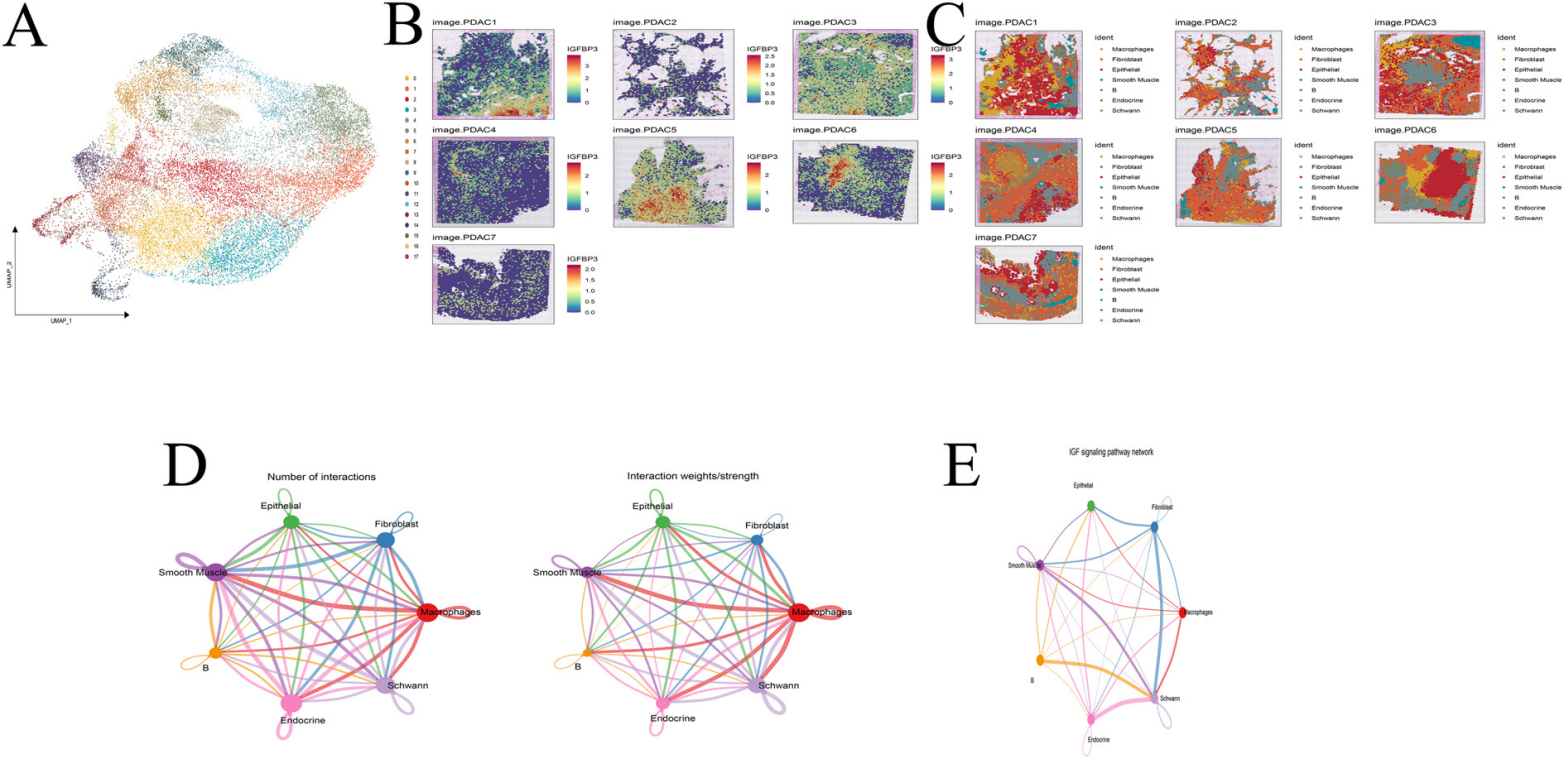
Spatial transcriptome analysis. **(A)** Cell clustering. **(B)** Expression of IGFBP3 across 7 samples. **(C)** Cell annotation. **(D, E)** Cell communication analysis. **(F)** IGF pathway analysis.

Following rigorous quality control, dimensionality reduction, and clustering, we identified 18 distinct cellular subpopulations within the spatial transcriptomic landscape (**Figure 10A**). IGFBP3 was consistently highly expressed across seven spatial samples (**Figure 10B**). Cell types identified in the single-cell dataset were projected onto the spatial transcriptomic data using Multimodal Intersection Analysis (MIA), allowing the localization of specific cell populations within tumor regions (**Figure 10C**). IGFBP3-high cells were predominantly localized in epithelial cells, primarily within the tumor core of PDAC. These IGFBP3-high regions exhibited significant enrichment of fibroblasts and B cells in their microenvironment. Cell–cell communication analysis revealed robust interactions between fibroblasts and Schwann cells via the IGF signaling axis (**Figures 10D, E**).

Among the IGFBP family members, IGFBP3 had the highest hazard ratio in univariate Cox regression (HR = 1.205), was highly expressed in high-risk patients, and was thus selected for further investigation.

**Figure 11:**
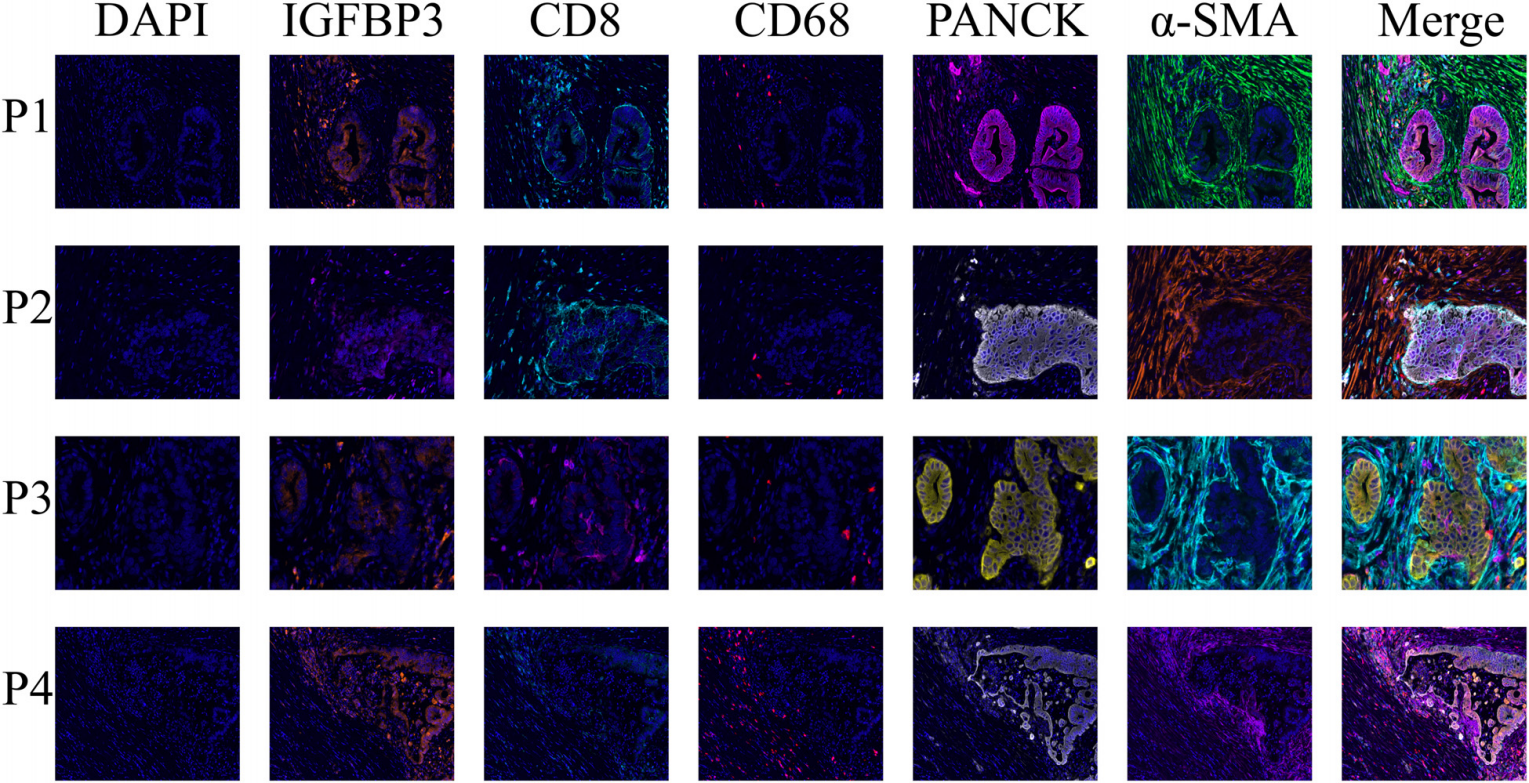
The multiplex immunohistochemistry staining demonstrated the distribution of IGFBP3 in the tumor immune microenvironment of PDAC, showing its presence in both cancer-associated fibroblasts and tumor cells.

Interestingly, we found a significant negative correlation between the risk score of the model and CD8+ T cell infiltration (**Figure 6A**, **Supplementary Figure S4**). Consistent with single-cell RNA sequencing and spatial transcriptomics data, mIHC confirmed the presence of IGFBP3 in both tumor cells and cancer-associated fibroblasts within the TME of PDAC (**Figure 11**). Since the spatial transcriptomics platform used in this research was the 10x Visium Cytassist version, with a resolution of 55μm, we conducted mIHC analysis to visually demonstrate the distance between high IGFBP3-expressing tumor cells and CD8+ T cells. Meanwhile, a higher expression of IGFBP3 indicates a worse prognosis (**Supplementary Figure S6C**) (HR 4.18 (95 CI 1.68-10.39)).

Furthermore, we designed a co-culture experiment, in which PANC-1 cells were co-cultured with CD8+ T cells. The results showed that after knocking down IGFBP3 in the PANC-1, the degree of CD8+ T cell infiltration significantly increased (**Supplementary Figure S6D**).

In terms of spatial distance, we statistically assessed the relationship between the expression of IGFBP3 in epithelial cells (PANCK) and the number of infiltrating CD8+ T cells within a 200μm radius (**Supplementary Figures S6A, B**). We found that tumor cells with high IGFBP3 expression were surrounded by significantly fewer CD8+ T cells (**Supplementary Figures S6A, B**). In addition, spatial transcriptome analysis also confirmed that CD8+T cells around tumor cells with high IGFBP3 expression were significantly reduced (**Supplementary Figure S7**). Taken together, these findings suggest that patients with high-risk scores based on the IGFBP3-based prognostic score could be categorized as the immune exhaustion group, indicating that these high-risk patients may have limited benefit from immune therapy.

## Discussion

The prognosis for pancreatic cancer remains exceedingly poor. A variety of prognostic markers have been developed, incorporating clinical parameters, laboratory indices, and molecular biomarkers (24, 29, 30). In this study, we systematically explored the role of the IGFBP family in PDAC through comprehensive bioinformatics analysis.

We first classified PDAC patients into two subtypes based on IGFBP family gene expression. Subtype A was associated with a worse prognosis. Notably, significant differences in immune cell infiltration and immune-related functions were observed between the subtypes. We further identified three gene-based subtypes, and correlation analysis revealed that these gene signatures could reflect both patient prognosis and the characteristics of the TME. On this basis, we constructed a prognostic model using differentially expressed genes. The model’s predictive performance was validated by survival analysis and ROC curves. Importantly, the risk score showed meaningful associations with TMB, immune-related signatures, clinical features, and drug sensitivity. We further developed a nomogram that integrated clinical variables and risk scores, demonstrating good performance in stratifying patients and supporting clinical decision-making.

Previous studies have highlighted the involvement of various IGFBP family members in the progression of PDAC. IGFBP1 has been found to be highly expressed in PDAC liver metastases, suggesting its potential as a clinical biomarker. Due to its role in tumor progression, IGFBP2 has also been studied extensively as a tumor biomarker (31). *Xu* et al. reported that plasma IGFBP2 levels in PDAC patients were significantly higher than those in patients with chronic pancreatitis, adenitis, or healthy individuals, and this elevation was associated with poor overall survival (32). *Huang* et al. demonstrated that pancreatic cancer cells secrete IGFBP3 to induce muscle atrophy, which may contribute to cancer cachexia in patients with advanced PDAC (33). IGFBP5, a potential regulator of cell proliferation, is overexpressed in PDAC and may play a critical role in the malignant transformation of normal pancreatic epithelial cells (34). In another study, *Han* et al. found that GSG2 knockout upregulated IGFBP6 expression, thereby inhibiting the proliferation, colony formation, and migration of pancreatic cancer cells (35). IGFBP7, which is downregulated in PDAC, functions as a tumor suppressor in various cancers. Its low expression has been linked to increased cellular proliferation and poor postoperative outcomes (36). In our study, we comprehensively integrated all IGFBP family genes to develop a prognostic model, offering new insights into their collective role in PDAC progression and prognosis.

Although the IGFBP family was initially believed to function merely as passive carriers of free IGFs, emerging evidence suggests that their biological roles extend beyond the endocrine transport of IGFs. Several IGFBPs have been reported to exhibit IGF-independent cellular functions, such as promoting cell migration without activating the IGF1 receptor (37). For instance, exogenous IGFBP3 has been shown to significantly inhibit the growth of human breast cancer cells through unique interactions with cell surface proteins (38). In pancreatic cancer, the abundant stromal compartment plays a pivotal role in regulating IGFBP expression (39). This stroma is rich in proteases capable of degrading IGFBPs, leading to elevated levels of free IGFs in the tumor microenvironment and consequently enhancing oncogenic IGF signaling (40). These biological insights underscore the rationale for incorporating IGFBP family genes into our prognostic model, aiding in the understanding of tumor biology in PDAC.

Tumor immune evasion is a well-recognized hallmark of cancer progression (41). The dynamic interaction between the immune system and tumor cells plays a crucial role in determining disease outcome (42). Factors such as tumor-infiltrating lymphocytes, TMB, and immune checkpoint receptor expression are considered key predictors of immunotherapy efficacy (43). The PDAC TME is characterized by high immunogenicity but low immunoreactivity, marked by inadequate immune activation and pronounced immune suppression (44). Immunotolerance can arise during any stage of tumor development and is maintained or intensified via various mechanisms, serving as a major obstacle to effective tumor immunity (45, 46). In our study, immune cell infiltration analysis revealed that immune activity was predominantly associated with the low-risk group. Furthermore, the risk score demonstrated a positive correlation with tumor purity and a negative correlation with mutational load. We also observed that several key immune checkpoint genes were upregulated in the low-risk group, suggesting a more immunogenic profile that may benefit more from immune checkpoint blockade therapies. These findings highlight the potential of our model to guide immunotherapy strategies in PDAC patients.

In addition, TMB has been reported as a predictive marker for response to immune checkpoint inhibitors (47). Mutations in TP53, often induced by carcinogenic exposures, are independently associated with poor clinical outcomes (48). Consistent with previous reports, our analysis showed a significantly higher TP53 mutation rate in the high-risk group, further reinforcing the model’s prognostic validity and its potential to inform treatment selection.

One of the long-standing challenges in PDAC treatment is drug resistance, particularly in advanced-stage disease. To address this, we investigated potential therapeutic agents based on the risk stratification provided by our prognostic model, aiming to improve drug sensitivity and treatment efficacy. Recent advances in single-cell sequencing and spatial transcriptomics have revolutionized cancer research by enabling high-resolution exploration of gene expression. In our study, we identified considerable heterogeneity in IGFBP gene expression at the single-cell level, revealing complex expression patterns within different cell populations. Spatial transcriptomics further demonstrated that IGFBP3 expression varies across tumor subregions and microenvironmental contexts, indicating its regulation by diverse cellular components.

Notably, IGFBP3 appears to be a promising therapeutic target due to its high expression in PDAC. IGFBP3 is the most abundant IGFBP in adult serum, yet its role in PDAC has not been fully elucidated. Our integrated analysis using TCGA and GEO datasets revealed that IGFBP3 exhibited the highest tumor-specific expression and prognostic relevance among all IGFBP family members. Subsequent evaluation of the tumor immune microenvironment showed that IGFBP3 is primarily expressed in tumor cells and cancer-associated fibroblasts. This finding is consistent with our single-cell sequencing data, which also revealed that IGFBP3-high tumor cells tend to be spatially distant from CD8^+^ T cells, implying potential involvement in immune evasion mechanisms.

Despite the promising findings, our study has certain limitations. The prognostic model was developed and validated using retrospective public datasets, and prospective clinical validation is needed to confirm its generalizability. Moreover, functional validation was limited to the cellular level. In future work, we aim to expand upon these findings through *in vivo* experiments and clinical studies to further substantiate the role of IGFBP3 and refine its application in PDAC management.

## Conclusion

Based on the IGFBP family, we developed a robust prognostic model for PDAC and further integrated clinical parameters to construct a nomogram, which demonstrated strong predictive performance. This model enables accurate estimation of patient prognosis and characterization of TME. Moreover, the insights gained from our study may provide a foundation for novel therapeutic strategies and contribute to the advancement of personalized treatment in PDAC.

## Data Availability

The original contributions presented in the study are included in the article/**Supplementary Material**. Further inquiries can be directed to the corresponding author.

## Supplementary material

Supplementary Figure 1 The flowchart of this study.

Supplementary Figure 2 Cluster analysis. Heatmap of cellular senescence genes using cluster analysis (k = 3-9).

Supplementary Figure 3 Cluster analysis. Heatmap of cellular senescence genes using cluster analysis (k = 2, 4-9).

Supplementary Figure 4 Immunoassay of the model.

Supplementary Figure 5 Single cell analysis. IGFBP family genes expression in 12 cell types.

Supplementary Figure 6 Tumor cells with high IGFBP3 expression were surrounded by significantly fewer CD8+ T cells. A and B, mIHC analysis demonstrated that there was a relatively low level of CD8+ T cell infiltration around the IGFBP3-high epithelium. C, Kaplan-Meier survival analysis suggested that a high expression of IGFBP3 was associated with a poor prognosis. D, Knocking down IGFBP3 in the Panc1 cell line significantly increased the infiltration level of CD8+ T cells.

Supplementary Figure 7 CD8+T cells around tumor cells with high IGFBP3 expression were significantly reduced.
