# Supplementary figures and images for: Integrated multi-omics profiling to establish an IGFBP-based prognostic score for pancreatic ductal adenocarcinoma: unraveling prognostic biomarkers, immune microenvironment crosstalk, and therapeutic implications
Source: Front Immunol. 2025 May 15;16:1600527. doi: 10.3389/fimmu.2025.1600527 (PMC12119506; doi:10.3389/fimmu.2025.1600527)

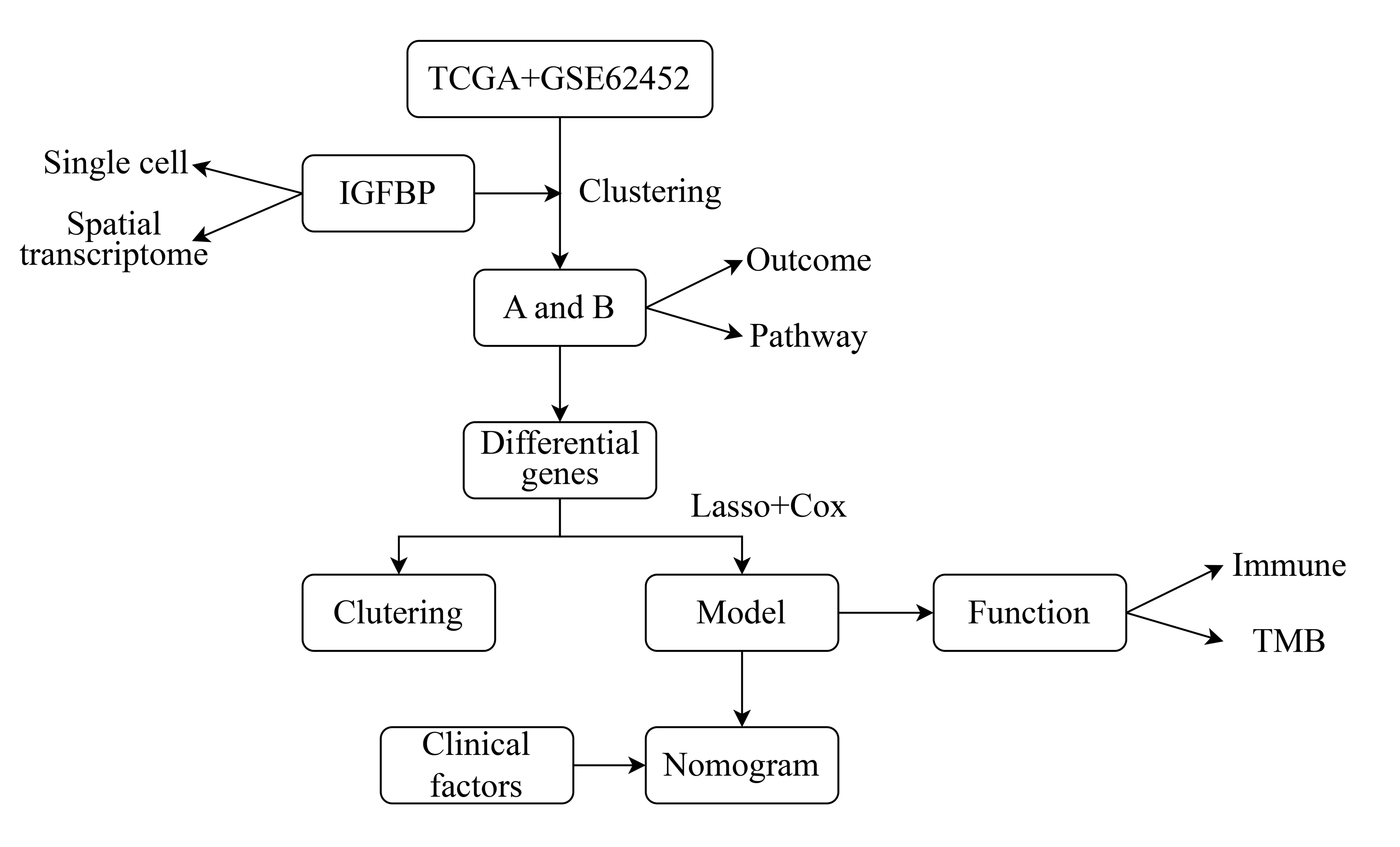

Supplement: Supplementary Figure 1 — The flowchart of this study. [file Image1.tif]

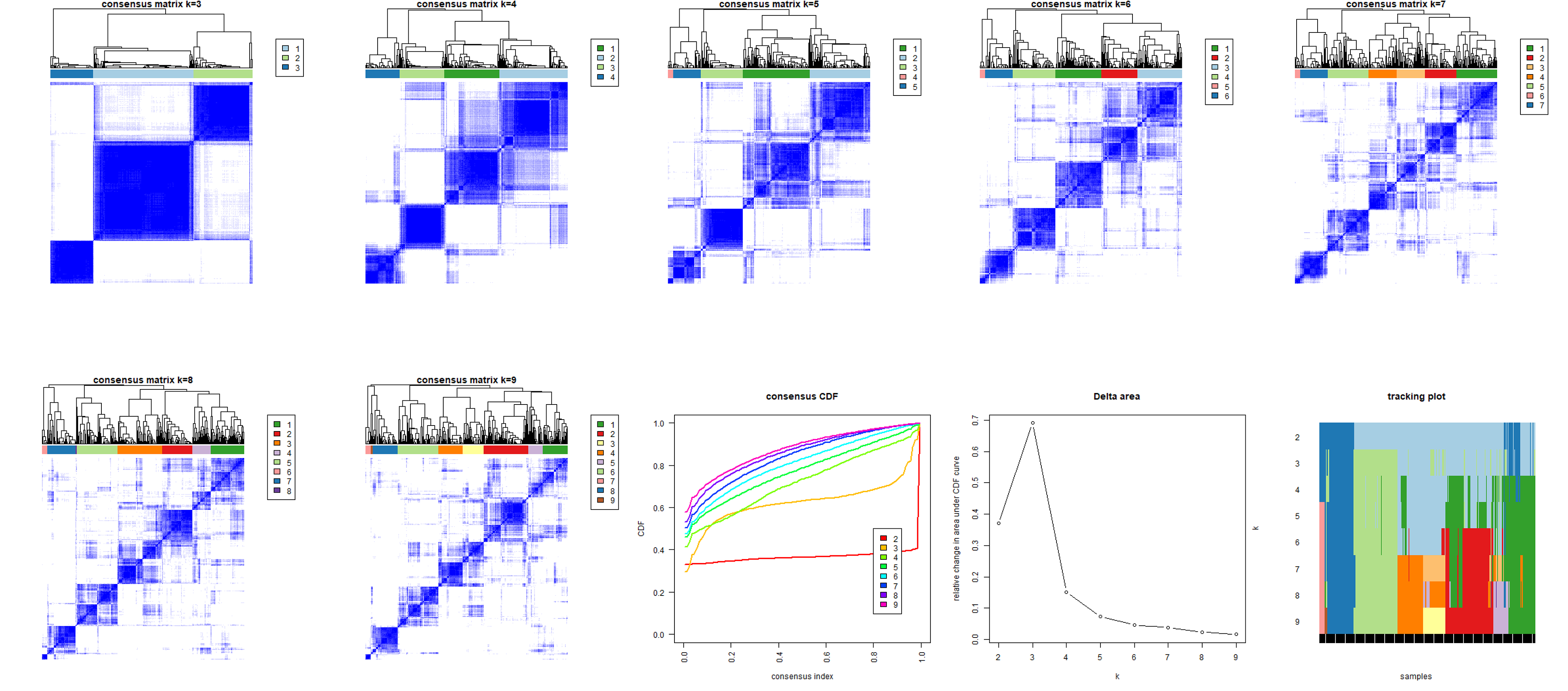

Supplement: Supplementary Figure 2 — Cluster analysis. Heatmap of cellular senescence genes using cluster analysis (k = 3-9). [file Image2.tif]

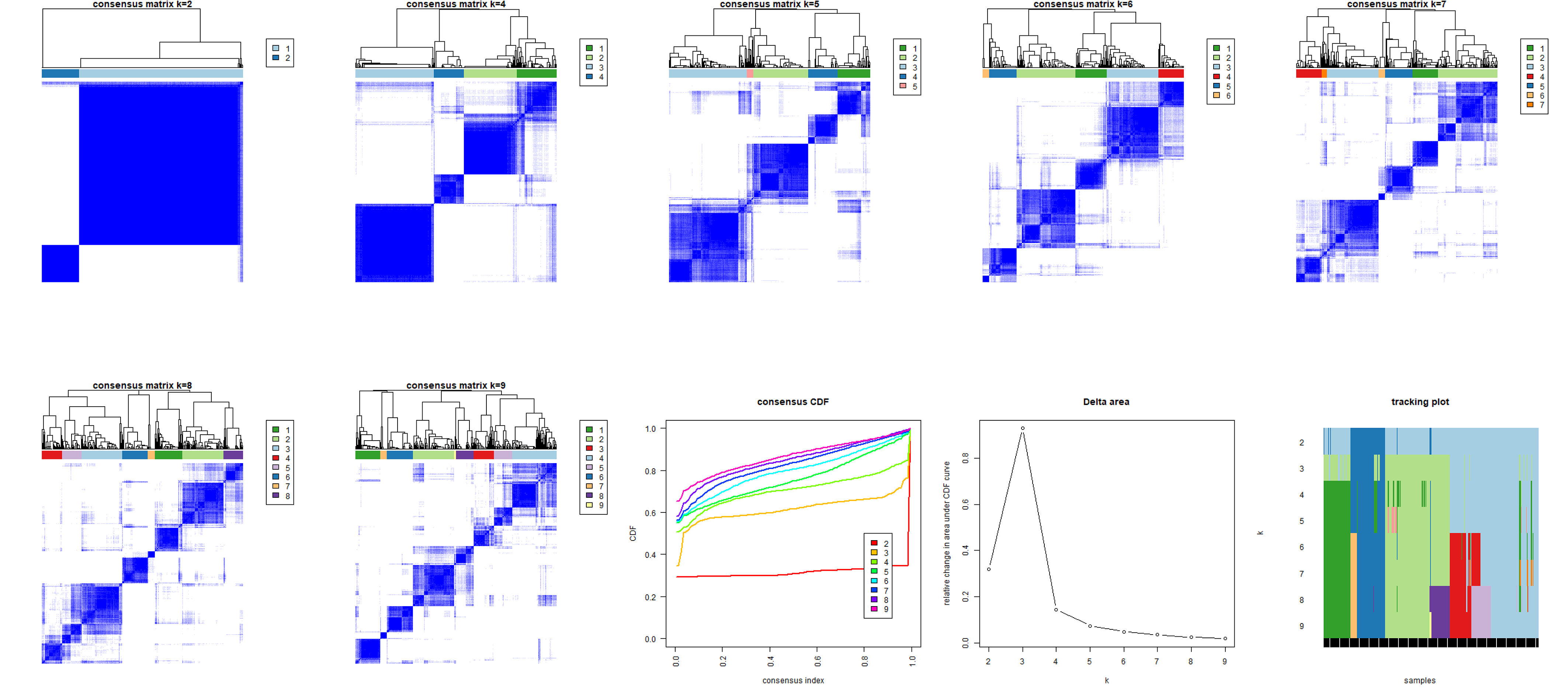

Supplement: Supplementary Figure 3 — Cluster analysis. Heatmap of cellular senescence genes using cluster analysis (k = 2, 4-9). [file Image3.tif]

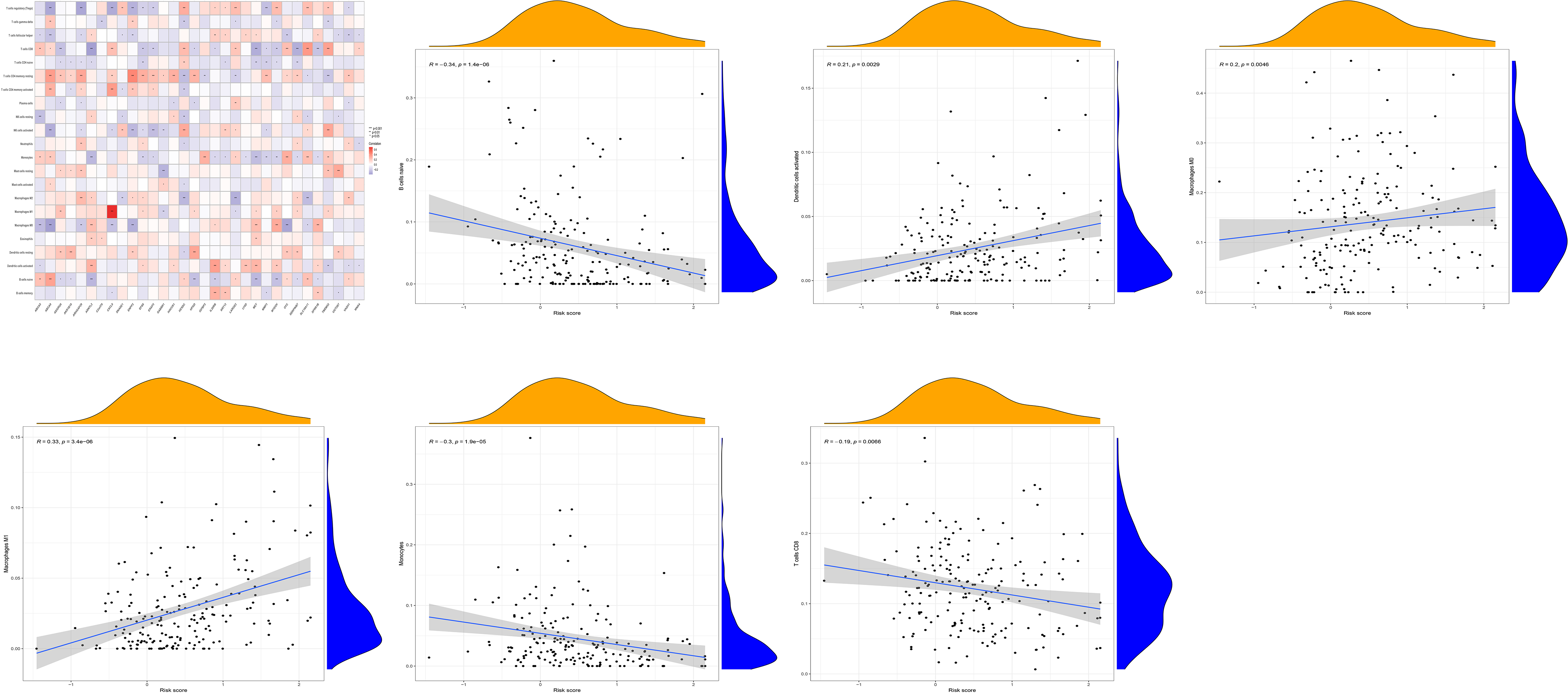

Supplement: Supplementary Figure 4 — Immunoassay of the model. [file Image4.tif]

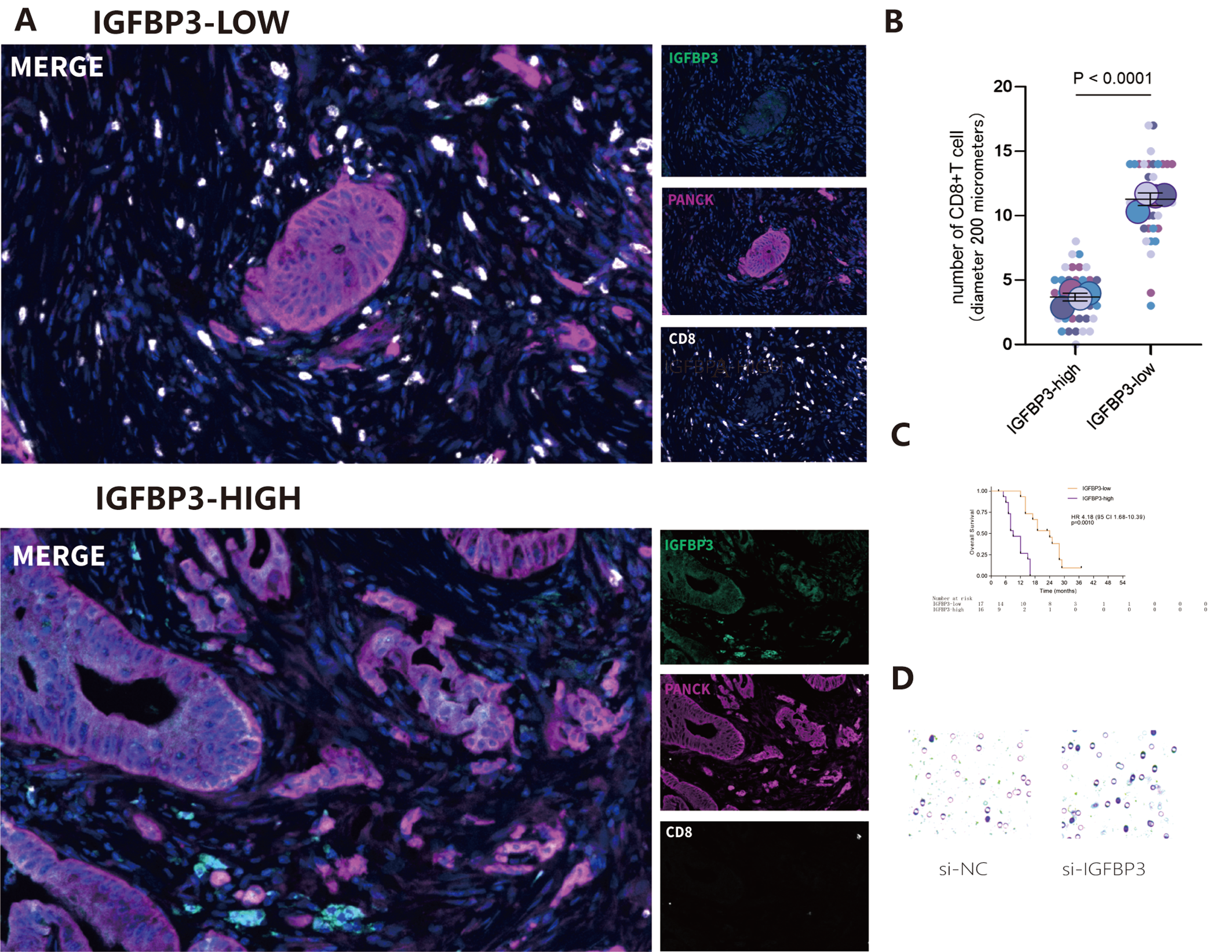

Supplement: Supplementary Figure 6 — Tumor cells with high IGFBP3 expression were surrounded by significantly fewer CD8+ T cells. A and B, mIHC analysis demonstrated that there was a relatively low level of CD8+ T cell infiltration around the IGFBP3-high epithelium. C, Kaplan-Meier survival analysis suggested that a high expression of IGFBP3 was associated with a poor prognosis. D, Knocking down IGFBP3 in the Panc1 cell line significantly increased the infiltration level of CD8+ T cells. [file Image6.tif]

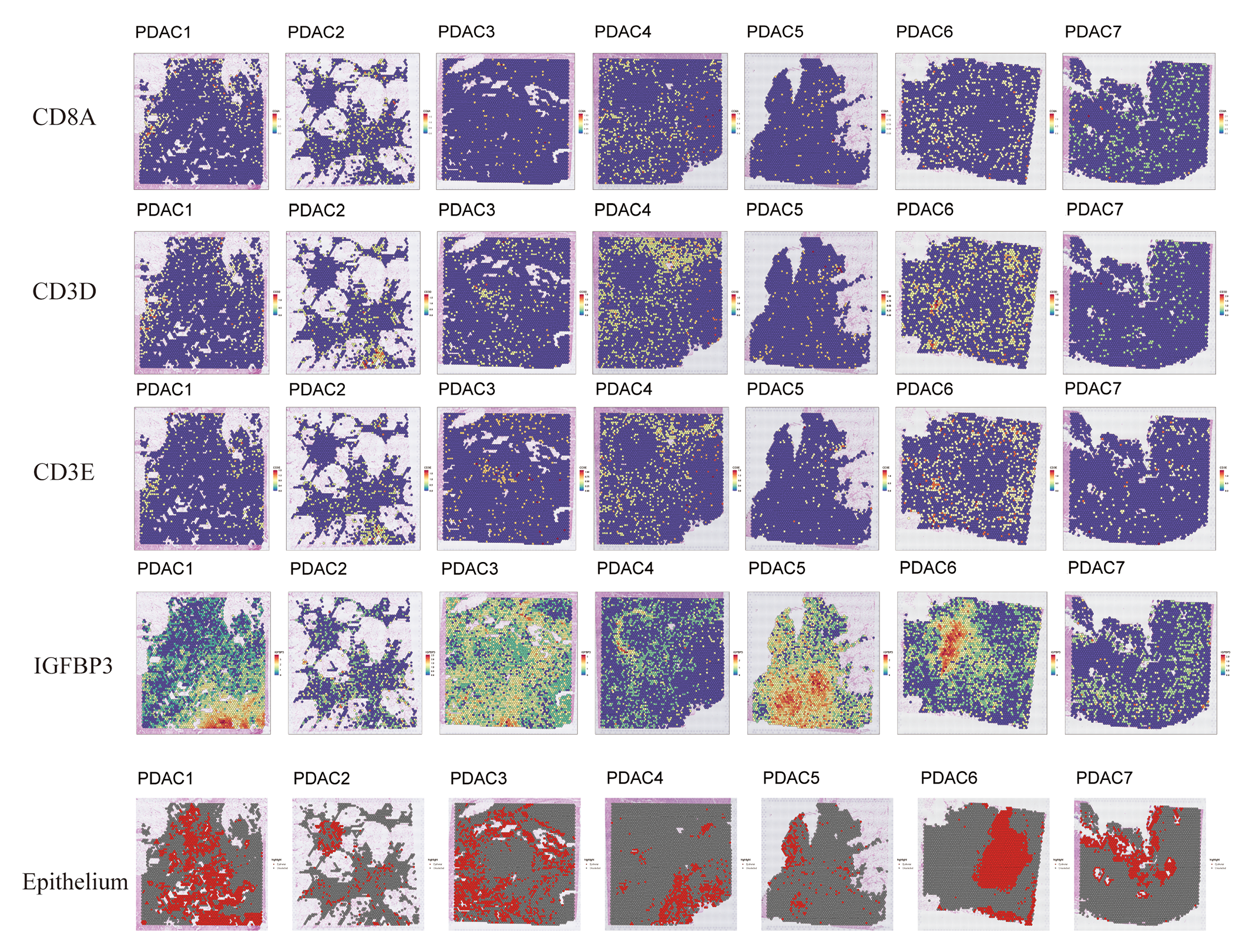

Supplement: Supplementary Figure 7 — CD8+T cells around tumor cells with high IGFBP3 expression were significantly reduced. [file Image7.tif]
